# The Coming Age of Flavonoids in the Treatment of Diabetic Complications

**DOI:** 10.3390/jcm9020346

**Published:** 2020-01-27

**Authors:** Teresa Caro-Ordieres, Gema Marín-Royo, Lucas Opazo-Ríos, Luna Jiménez-Castilla, Juan Antonio Moreno, Carmen Gómez-Guerrero, Jesús Egido

**Affiliations:** 1 Research Discovery and Innovation Department, FAES FARMA, S.A, Department of Physiology, Faculty of Medicine and Nursing, University of the Basque Country, 48940 Leioa (Bizkaia), Spain; tcaro@faes.es; 2 Renal, Vascular and Diabetes Research Laboratory, IIS-Fundación Jiménez Díaz, Universidad Autonoma de Madrid, Spanish Biomedical Research Centre in Diabetes and Associated Metabolic Disorders (CIBERDEM), 28040 Madrid, Spain; gmarinroyo@gmail.com (G.M.-R.); lucasopazo78@gmail.com (L.O.-R.); luna.jimenez@quironsalud.es (L.J.-C.); Cgomez@fjd.es (C.G.-G.); 3 Department of Cell Biology, Physiology and Immunology, University of Córdoba, 14004 Córdoba, Spain; juanmorenoguti@gmail.com; 4 Maimonides Biomedical Research Institute of Cordoba (IMIBIC), University of Córdoba, 14004 Córdoba, Spain; 5 Hospital Universitario Reina Sofía, 14004 Córdoba, Spain

**Keywords:** flavonoids, diabetes, microvascular complications, cardiovascular disease, diabetic nephropathy, oxidative stress, inflammation, therapeutics

## Abstract

Diabetes mellitus (DM), and its micro and macrovascular complications, is one of the biggest challenges for world public health. Despite overall improvement in prevention, diagnosis and treatment, its incidence is expected to continue increasing over the next years. Nowadays, finding therapies to prevent or retard the progression of diabetic complications remains an unmet need due to the complexity of mechanisms involved, which include inflammation, oxidative stress and angiogenesis, among others. Flavonoids are natural antioxidant compounds that have been shown to possess anti-diabetic properties. Moreover, increasing scientific evidence has demonstrated their potential anti-inflammatory and anti-oxidant effects. Consequently, the use of these compounds as anti-diabetic drugs has generated growing interest, as is reflected in the numerous in vitro and in vivo studies related to this field. Therefore, the aim of this review is to assess the recent pre-clinical and clinical research about the potential effect of flavonoids in the amelioration of diabetic complications. In brief, we provide updated information concerning the discrepancy between the numerous experimental studies supporting the efficacy of flavonoids on diabetic complications and the lack of appropriate and well-designed clinical trials. Due to the well-described beneficial effects on different mechanisms involved in diabetic complications, the excellent tolerability and low cost, future randomized controlled studies with compounds that have adequate bioavailability should be evaluated as add-on therapy on well-established anti-diabetic drugs.

## 1. Introduction

Diabetes mellitus (DM) is currently one of the greatest public health challenges in the world. Despite the new tools of control and prevention for early diagnosis, its prevalence is still increasing. Successive reports generated by the International Diabetes Federation have shown the increasing incidence of people with DM (actually 463 million adults) and the estimated projection for the year 2045 will rise to 700 million people affected [1].

It has been estimated that one out of every two individuals with DM is not aware of having the disease. This is particularly important in patients with type 2 diabetes (T2D) due to the long asymptomatic phase, which increases the risk of microvascular and macrovascular complications, the risk of death compared with normoglycemic individuals, and the tremendous economic cost for the health system [1,2,3,4]

The incidence of diabetes-related complications has declined substantially in the past decades worldwide probably due to a substantial improvement in preventive care programs for adults with diabetes and the extensive use of drugs controlling blood glucose, hypertension and dyslipidemia, among others [5]. However, a large burden of disease persists because of the continued increase in the prevalence of diabetes, mainly due to the threats of increasing obesity, world overpopulation and aging [6,7].

Although the recent availability of novel anti-diabetic drugs (e.g., incretin modulators and sodium-glucose transport protein-2 inhibitors) will certainly further decrease the rates of cardiovascular risk and mortality in diabetic patients, the research for new drugs to halt the progression of diabetic complications is still necessary [8,9,10,11]. Due to the complexity of the pathogenic mechanisms driving long-term complications of diabetes, including inflammation and oxidative stress, the clinical introduction of novel drugs combating those conditions would be of interest [12].

In this review, we have focused on the potential beneficial effects of flavonoids on different mechanisms involved in diabetic complication, as well as the current state of research related to the preclinical and clinical usefulness of these compounds in this clinical condition. On the whole, we provide information supporting the future development of well-designed clinical trials with flavonoids that have adequate bioavailability as add-on therapy on well-established anti-diabetic drugs.

## 2. Pathogenesis of Diabetic Complications

DM is associated with several chronic complications, mainly classified as macrovascular and microvascular complications. Among them, coronary artery disease, peripheral arterial disease and stroke are described as macrovascular complications, due to a close relationship with atherosclerotic mechanisms. On the other hand, diabetic microangiopathies or microvascular complications are described at the ocular (retinopathy), renal (nephropathy) and peripheral nerve (neuropathy) levels, and are characterized by changes in the thickness of the capillary basement membrane [13,14].

In the genesis of the diabetic complications, hyperglycemia is the driving force for clinical features, which in synergy with other risk factors (hypertension, obesity, dyslipidemia) potentiates and accelerates the histopathological features classically observed in each of the tissue-specific manifestations of the DM [15,16]. Simultaneously, as a consequence of hyperglycemia, alterations in signaling and glucose metabolism are observed, promoting insulin resistance in target organs and beta cell dysfunction [17].

The influence of the genetic background as an individual risk factor is not only important in type 1 diabetes (T1D), but rather in all tissue-specific manifestations of DM [18]. The analyses of multicenter population genetic studies and genome-wide association studies (GWASs) have reported certain risk polymorphisms (single-nucleotide polymorphisms—SNPs) that could be involved in the diverse pathogenesis of complications associated with DM [19]. The proposed actions for these SNPs are related to phagocytosis, cytoskeleton rearrangement, fibroblast migration and lymphocyte infiltration [20]. Other studies have suggested the influence of gene alterations associated with transport and glucose metabolism, the renin angiotensin aldosterone system (RAAS), transcription factors and growth factors [21,22].

The maintenance of a hyperglycemic state involves the anabolic-catabolic production of toxic metabolites, which activate metabolic and hemodynamic pathways through the formation of advanced glycation end products (AGEs), activation of protein kinase C (PKC) and polyol sorbitol pathway, activation of RAAS, vascular endothelial growth factor A (VEGFA) and endothelin-1 and receptor type A (ET1/ETA). These processes cause remodeling in the architecture of the different target organs and ultimately atrophic changes [23,24,25,26]. On the other hand, the presence of oxidative stress and altered redox homeostasis in resident cells are characteristics commonly observed in DM patients [27,28,29]. Uncoupling endothelial nitric oxide synthase (eNOS) and the bioavailability of nitric oxide (NO) are two main factors responsible for the changes of vascular reactivity and production of reactive oxygen and nitrogen species (ROS/RNS) [30,31,32]. Additionally, dysregulation between pro-oxidant enzymes (e.g., NADPH oxidase complex (Nox), xanthine oxidase, cytochrome 450 and myeloperoxidase) and antioxidant enzymes (e.g., superoxide dismutase, glutathione peroxidase and catalase) is a major contributor to redox imbalance [33,34,35].

Kinases and transcription factors involved in many inflammatory and oxidative stress responses, activate intracellular signaling pathways that lead to the production of pro-inflammatory, pro-oxidant and pro-angiogenic factors such as cytokines, chemokines, pro-oxidant enzymes, growth factors, adhesion molecules and extracellular matrix proteins, among others [36,37,38,39,40].

Chronic hyperglycemia is the main pathogenic factor involved in DM complications, mainly triggering systemic and local microinflammation and therefore an important therapeutic target to hamper the development and progression of diabetic complications [41].

## 3. General Aspects of Flavonoids

Flavonoids are an important class of natural products; more precisely, flavonoids are one of the largest groups of plant secondary metabolites, comprising at least 6000 phenolic compounds. They are widely found in fruits, vegetables, nuts, grain seeds, cocoa, chocolate, tea, soy, red wine, herbs and beverage products [42,43,44,45].

**Figure 1:**
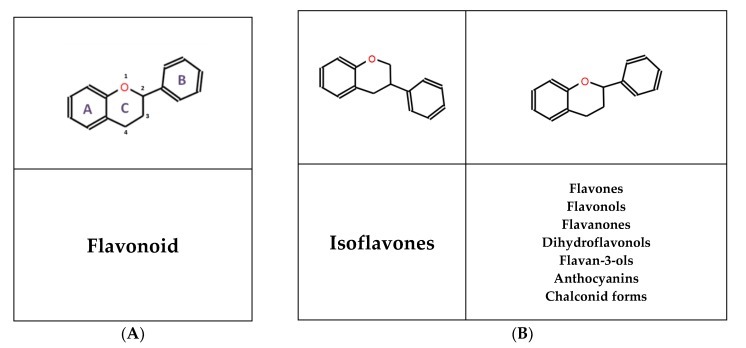
(**A**) Common chemical structure of flavonoids. (**B**) Chemical structure of flavonoid subtypes described in the literature.

Flavonoids share a common chemical structure (Figure 1A) based on a C6-C3-C6 skeleton, where two aromatic rings are linked by a three-carbon chain that forms an oxygenated heterocyclic ring [42,43,44]. Depending on the degree of unsaturation and the substitution pattern, different flavonoid classes can be distinguished (Figure 1B): *flavones, flavonols, flavanones, flavan-3-ols, anthocyanins, dihydroflavonols, and isoflavones,* as well as the biogenetic intermediate *chalconoid forms* [43].

Flavonoids have long been used in traditional medicine mainly due to their antioxidant properties; for example, *Radix Puerariae* (*Puerarin*) has been used to treat diabetes for thousands of years. Flavonoids are associated with a broad spectrum of health-promoting effects because of their anti-oxidative, anti-inflammatory and anti-carcinogenic properties and their capacity to modulate key cellular enzyme functions [46]. Nowadays, flavonoids are present in the pharmaceutical market in different formats such as tablets, hard capsules, granules, oral solutions and gel. The main commercialized flavonoids (*Diosmin, Hesperidin, Troxerutin and Hidrosmin*) are mostly used for the treatment of edema and venous insufficiency [47].

## 4. Recent Preclinical Advances in the Anti-Diabetic Actions of Flavonoids

The anti-inflammatory, anti-oxidant and anti-hyperlipidemic effects of flavonoids have been widely demonstrated. For this reason, there is much interest in these compounds as potential anti-diabetic drugs. This is reflected in the large number of in vitro and in vivo studies carried out to assess the effect of flavonoids in the context of diabetes [48,49,50].

Flavonoids modulate glucose metabolism or insulin sensitivity at different levels, increasing glucose uptake and insulin secretion, and inhibiting glucose production [51]. There are several publications describing the anti-diabetic action of flavonoids on different tissues such as skeletal muscle, white adipose tissue, liver, small intestine, pancreas and kidney [52]. In skeletal muscle and white adipose tissue, flavonoids mainly act on glucose transporter type 4 (GLUT4), increasing its translocation to the plasma membrane allowing the uptake of glucose from blood. The translocation of GLUT4 can be mediated by activation of different pathways such as phosphoinositide 3-kinase (PI3K)/protein kinase B (Akt) [53], and AMP-activated protein kinase (AMPK) [54]. In skeletal muscle, Yamashita et al. demonstrated that *liquorice* flavonoid oil promoted GLUT4 translocation by activation of the AMPK pathway and improved symptoms in T2D [54]. *Sea buckthorn extract*, rich in *quercetin*, *isorhamnetin* and *gallic acid*, also stimulated GLUT4 translocation in adipocytes and improved glucose uptake activity [53].

On the other hand, some flavonoids act by inhibiting the expression or translocation of GLUT2). In liver, *Baicalin* reduces GLUT2 in addition to other gluconeogenic genes, such as glucose-6-phosphatase (G6Pase) and phosphoenolpyruvate carboxykinase (PEPCK), therefore inhibiting the synthesis of glucose and its subsequent release into the bloodstream [55]. The production of hepatic glucose is also inhibited by other flavonoids such as *epigallocatechin gallate*, that acts by reducing the expression of PEPCK and G6Pase in hepatic (HepG2) cells [56,57] and *xanthohumol*, that inhibits GLUT2 expression in the liver of diabetic rats [58]. The reduction of GLUT2 was noted, not only in liver but also in human intestinal cells, by the administration of *anthocyanin-rich berry extract* [59], *myricetin*, *fisetin* and *quercetin* [60].

An effective strategy to reduce increased glucose levels (in postprandial hyperglycemia, for example) is to retard the intestinal digestion of complex carbohydrates through the inhibition of α-glucosidases. In this sense, flavonols such as *rutin*, *quercetin* and *isoquercetin* are considered α-glucosidase inhibitors. The inhibition of α-glucosidase prevents glucose absorption and consequently reduces postprandial blood glucose levels in the Sprague–Dawley rat model [61].

Another beneficial effect that flavonoids exert is the preservation of β-cell viability by reducing inflammation and oxidative damage. The capacity of *guava leaf extract* (containing *anthocyanins* and *quercetin*) to increase the pancreatic levels of different antioxidant enzymes such as superoxide dismutase (SOD), catalase (CAT) and glutathione peroxidase (GPx) and to downregulate the expression of inflammatory mediators, such as TNF-α and IL-6, in streptozotocin (STZ)-induced diabetic rats has been demonstrated [62]. *Naringin* also has a protective effect on β-cells due to its capacity to prevent apoptosis through the inhibition of both the intrinsic (mitochondria-mediated) and extrinsic (death receptor-mediated) pathways. These anti-apoptotic effects are associated with a reduction in ROS and inflammatory cytokines [63].

**Figure 2:**
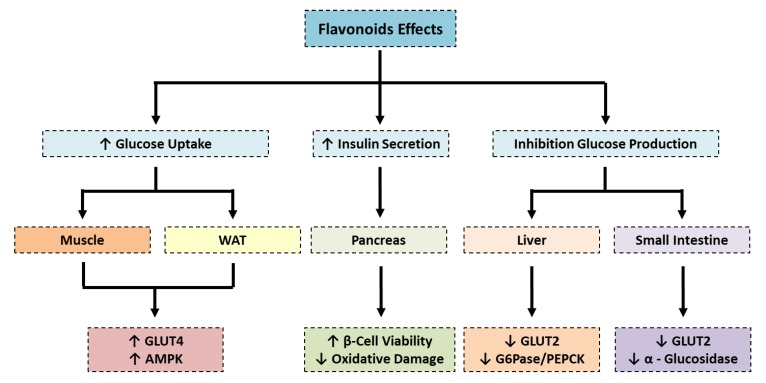
Effect of flavonoids on glucose metabolism. The main effect of flavonoids on skeletal muscle and adipose tissue is the enhancement of glucose uptake mediated by the translocation of GLUT4 to the plasmatic membrane. Conversely, in the liver, flavonoids act in a different way. They try to reduce glucose blood levels both by reducing gluconeogenic genes (such as G6Pase and PEPCK) and therefore glucose production and GLUT2, and therefore preventing the release of glucose from liver to the bloodstream. The production of glucose is also prevented in the intestine by blocking the digestion of complex carbohydrates. In the pancreas, flavonoids predominantly reduce oxidative stress improving the viability of β-cell, consequently ameliorating insulin secretion. WAT: White adipose tissue; AMPK: AMP-activated protein kinase; GLUT4: glucose transporter type 4, GLUT 2: glucose transporter type 2, G6Pase: glucose-6-phosphatase, PEPCK: phosphoenolypyruvate carboxykinase.

In summary, flavonoids exert their anti-diabetic effects in part by acting on different tissues (pancreas, liver, adipose tissue and skeletal muscle) involved in the modulation of glucose homeostasis and insulin sensitivity (Figure 2).

## 5. Current Status of Experimental Research on Flavonoids in Chronic Complications of Diabetes

Due to the potent antioxidant effect of flavonoids, the therapeutic potential of these compounds has been evaluated in several chronic diseases including cancer, neurodegenerative and cardiovascular disorders, and diabetes. Thus, the present review focuses on the most recent (last 3 years) preclinical studies of flavonoids for the treatment of microvascular (nephropathy, retinopathy and neuropathy) and macrovascular (cardiovascular) complications of diabetes (Table 1).

### 5.1. Diabetic Nephropathy

Diabetic nephropathy (DN), one of the most prevalent complications in diabetes, is characterized by a progressive development of renal dysfunction. In the early stage, the first symptom is microalbuminuria (appearance of low levels of albumin in the urine, 30–300 mg/g), which precedes macroalbuminuria (high levels of albumin in the urine, >300 mg/g), leading to a deterioration of renal function and progression to End Stage Renal Disease (ESRD) [64]. Accumulating evidence has clearly demonstrated that immune-inflammatory response plays a paramount role in the onset and progression of diabetic kidney disease. This process is mediated by elements of the immune system (e.g., lymphocytes and monocytes/macrophages) and effector molecules including cytokines, growth factors, chemokines adhesion molecules, and enzymes [65]. Several in vitro and in vivo studies have evaluated the role of flavonoids on DN, most of them reporting a positive effect on renal function, evidenced by reduced levels of serum creatinine and urine albumin [66,67]. For example, in a rat model of STZ-induced DN combined with high-fat, high-carbohydrate diet, the treatment with *mangiferin* extracted from *Pyrrosiae folium* down-regulated IL-6, TNF-α and IL-1β expression and improved renal pathological damage [67]. Similarly, *corn silk extract*, *liquiritigenin* and *naringin* also exhibited anti-inflammatory activity by reducing the expression of IL-1β and IL-18 in mesangial cells exposed to high glucose conditions [68,69,70].

In diabetic kidney, high levels of glucose and other metabolites, including inflammatory cytokines and free fatty acids, lead to an excessive ROS production, being Nox and mitochondrial dysfunction the main sources of ROS generation [71]. It has been demonstrated that ROS promote serine phosphorylation of insulin receptor and insulin receptor substrate through different pathways inducing insulin signaling suppression [72]. Moreover, overproduction of ROS modulates activation of PKC, mitogen-activated protein kinases (MAPK), and several transcription factors and cytokines responsible of the inflammatory response and extracellular matrix accumulation with further progression to ESRD [71].

A number of studies have described the renal antioxidant effects of several flavonoids. *Formonetin* reduced oxidative stress burden and attenuated kidney damage in a T2D rat model by increasing the activity of the antioxidant enzymes SOD and CAT, along with induction of the protective molecule Sirtuin-1 [73]. *Diosmetin*, a flavonoid extracted from the leaves of *Olea europaea* with strong antioxidant property, restored the reduced levels of SOD and NO observed in the kidney of diabetic rats and decreased the levels of malondialdehyde (MDA), a lipid peroxidation marker [66]. The flavanone *naringin* also attenuates ROS production in diabetic kidneys with concomitant reduction in Nox4 expression and increase in antioxidant enzymes SOD and GPx [74]. In vitro experiments have also demonstrated that the strong anti-oxidant effect of different flavonoids (e.g., *corn silk extracts* and *liquiritigenin*) is linked to Nox4 inhibition and SOD induction [68,69,75].

AGEs could accumulate in glomerular basement membrane, podocytes, endothelial cells and mesangial cells in diabetic patients compromising renal function through different mechanisms, such as oxidative stress, chronic inflammation and fibrosis. Therefore, the blockade of AGEs and their receptor system (RAGEs) as well as downstream related pathways has become another therapeutic target for the treatment of DN [76]. In this regard, the protective effect of *kaempferitrin* was evaluated in glomerular mesangial cells exposed to AGEs [75]. In this study, *kaempferitrin* reduced AGE-induced oxidative stress by increasing SOD activity and by reducing MDA levels and ROS generation. Moreover, this flavonoid prevented the increase in collagen IV and TGF-β1 levels [75]. The anti-fibrotic effect has also been demonstrated by other flavonoids such as *chrysin* which inhibited AGEs-induced expression of collagens, and matrix metalloproteinases in mesangial cells and prevented collagen and AGEs accumulation in diabetic kidneys from the *db/db* mouse model of T2D [77].

The polyphenolic natural product *resveratrol* though described as a non-flavonoid compound, also reduced AGE levels in kidneys, attenuating the progression of DN [78].

### 5.2. Diabetic Retinopathy

Diabetic retinopathy (DR) is a complication of diabetes affecting the eye that may cause severe vision loss and blindness. High glucose levels are associated with damage to the vasculature of retina. Diabetic macular edema is a consequence of DR that causes swelling in the area of the retina called the macula [79].

Several studies have assessed the potential benefits of flavonoids on eye affectation during diabetes. Most of them have shown the antioxidant, anti-inflammatory and anti-angiogenic effect exerted by flavonoids in the eye, leading to an improvement in DR. For example, *catechin* inactivated the NF-κB signaling pathway and reduced the expression of downstream target genes (e.g., IL-1β, IL-6, and TNF-α) in the retina of STZ-induced diabetic rats [80]. *Biochanin A* showed a dual action on inflammation and angiogenesis in a type 1 diabetic rat model, associated with decreased expression of TNF-α, IL-1β and VEGF in the retina [81]. Similarly, administration of *morus alba* extract [82] or the flavanone *naringenin* [83] attenuated oxidative, apoptotic, angiogenic and neurodegenerative markers, whereas *gallangin* [84] attenuated inflammation and blood–retinal barrier breakdown in diabetic rats.

An in vitro study performed in human retinal capillary endothelial cells exposed to high glucose concentrations demonstrated that *blueberry anthocyanin* extracts reduced VEGF by inhibiting the Akt pathway. This anti-angiogenic effect was accompanied by an improvement in the oxidative and inflammatory state, evidenced by increased antioxidant enzymes (SOD and CAT) and reduced pro-oxidant enzymes (Nox4 and eNOS) and NF-κB activity [85]. Moreover, anti-inflammatory and anti-oxidant effects have also been demonstrated by *myricetin*, *eriodyctiol* and *galangin* in retinal epithelial, ganglial and microglial cells, respectively [84,86,87]. In addition, *baicalin* protects human retinal pigment epithelial cells against hyperglycemic conditions by reducing the proliferative, anti-apoptotic and anti-inflammatory effects by up-regulation of miR-145 and further inhibition of NF-κB and p38 MAPK pathways [88].

Kang et al. demonstrated that *chrysin* ameliorated the malfunction of the retinoid visual cycle by increasing retinoid binding proteins (RPE65, LRAT, RDH5, and rhodopsin), downregulating angiogenic factors, and blocking AGE-induced endoplasmic reticulum stress in glucose-stimulated RPE cells and diabetic retina of *db/db* mice [89].

### 5.3. Diabetic Neuropathy

Diabetic neuropathy is a peripheral nerve disorder that affects organs which are innervated by the autonomic nervous system like heart, eyes, gastrointestinal tract, kidney, and liver. Diabetic neuropathy affects more than 50% of all diabetic patients and is commonly associated with loss of sensation in the feet leading to difficulty to walk and weakness in the foot muscles [90]. Oxidative stress and inflammation play a central role in neuronal degeneration. Oxidative stress leads to activation of inflammatory and apoptotic pathways resulting in insufficiency of neurochemical growth factors, neurovascular dysfunction, demyelination of neurons and increased autoimmune damage [91,92]. Therefore, the inhibition of oxidative stress and inflammation cascades could be an option to prevent the development of diabetic neuropathy.

Several examples of the protective actions of flavonoids against diabetic neuropathy have been published. *Catechin*, in a model of STZ-diabetic induced rats, was able to reduce the neuronal damage by increasing SOD and CAT, and reducing MDA and lymphocyte infiltration in nerve tissues [93]. *Deguelin* also ameliorates diabetic neuropathy, attenuated oxidative stress and neuroinflammation through the activation of NRF2 pathway, and partially restored the conduction velocities of neurons in diabetic rats by increasing (Na^+^-K^+^) ATPase activity [94]. A similar neuroprotective effect against oxidative stress and inflammation was reported for *phloretin*, which either alone or in combination with the anti-neuropathic agent duloxetine promoted nerve fiber regeneration in STZ-induced diabetic rats [95].

A study performed to assess the role of *resveratrol* in diabetic neuropathy showed that it provides neuroprotection by increasing SOD and reducing GSH and inhibiting nitrosative stress and myeloperoxidase (MPO) activity in diabetic rats [96]. Likewise, *quercetin* in combination with moderate exercise training attenuated neuropathy and restored histological alterations in sciatic nerves in STZ-diabetic rats through antioxidant enzyme induction and down-regulation of free radical production [97]. In vitro experiments revealed that inhibition of NF-κB and activation of NRF2/Heme-oxygenase-1 (HO-1) signaling pathways contribute to the anti-inflammatory and antioxidant effects of *quercetin*, *cinnamaldehyde* and *hirudin* in dorsal root ganglion neurons exposed to high glucose [98,99]. Similarly, *puerarin* inhibited oxidative stress and apoptosis induced by hyperglycemia in Schwann cells via suppression of caspase-3 cascade [100].

### 5.4. Diabetic Macrovascular Complications

Macrovascular manifestations of diabetes include primarily diseases of the coronary and peripheral arteries, leading to cardiovascular events. Early macrovascular disease is associated with atherosclerotic plaque in the vessels that supply blood to the heart, brain, limbs, and other organs, whereas late stages of macrovascular disease involve complete arterial obstruction whit an increased risk of myocardial infarction and stroke [15].

It is well known that DM increases cardiovascular morbidity and mortality. However, surprisingly in the last years, the role of flavonoids on cardiovascular complications associated with DM has been poorly studied compared to other diabetic complications such as retinopathy or nephropathy. Most of the studies are focused on assessing the role of flavonoids on endothelial dysfunction since it is one of the earliest and most prevalent abnormalities of cardiovascular complications associated with DM [101].

Bharat et al. tested the possible beneficial effect of *blueberry anthocyanins* in human aortic endothelial cells exposed to palmitic acid. The authors found that *anthocyanins* attenuated endothelial dysfunction through the suppression of Nox-mediated ROS production [102]. *Rutin* also prevented endothelial dysfunction under hyperglycemic conditions both in vitro (human umbilical vein endothelial cells) and in vivo (carbohydrate diet-treated rats), through inhibiting Nox4-mediated ROS generation and NLRP3 inflammasome activation [103].

In a mouse model of T2D, *liquiritigenin* attenuated cardiac injury through its anti-fibrotic and anti-inflammatory effect by inactivating NF-κB signaling pathway [104]. Likewise, *apigenin* protected from cardiovascular damage by preventing cardiomyocyte hypertrophy and fibrosis and attenuating oxidative and inflammatory state in experimental T1D [105].

*Resveratrol* was able to reduce ROS production in human aortic endothelial cells through the modulation of autophagy as a result of AMPK-mTOR pathway regulation [106] and also exhibited cardioprotective effects *in vivo*, as it restored the disruption and thickening of the muscle fibers in a diabetic rat model with coronary heart disease [107]. Moreover, *resveratrol* in combination with glibenclamide was able to reduce ischemia/reperfusion induced arrhythmias in a diabetic rat model [108].

On the other hand, lipid alterations are very common in patients with diabetes, mainly among those who have T2D. It is well known that there is a close relation between hyperlipidemia and the outcome of cardiovascular events in the context of diabetes. Specifically, alterations on the lipid profile can eventually result in atherosclerosis [109]. Therefore, an adequate control of lipid levels is necessary to prevent diabetic complications. Considering the relevance of flavonoids in recent years in the study of diabetes, it is not surprising the existence of numerous studies confirming the anti-hyperlipidemic effect of these compounds. In this sense, a study performed by Mohammed Yusof demonstrated that *Hibiscus sabdariffa* (*roselle*) polyphenol-rich extract, including *anthocyanidins*, acts as a cardioprotective agent by lowering total cholesterol, triglyceride and LDL and increasing HDL [110]. Likewise, *isoquercertin* [111] and *galangin* [112] have been shown to decrease triglycerides, phospholipids and free fatty acids in diabetic rats, whereas scutellarin alleviated dyslipidemia in *db/db* mice by increasing HDL and reducing triglycerides and total cholesterol [113].

## 6. Clinical Studies on Flavonoids in Diabetes

In contrast to the extensive experimental research in various models of diabetic complications, a limited number of published clinical studies of flavonoids in diabetic patients are available (Table 2 and Table 3).

### 6.1. Diabetic Nephropathy

Several studies have provided evidence supporting a beneficial effect of flavonoids, flavonoid-rich foods and polyphenolic natural product on DN. To date, the main bioactive phytochemicals assayed in clinical trials on DN are: *silymarin*, *green tea extract*, *soy protein* and *curcumin*. Fallahzadeh et al. [114] evaluated the renoprotective effect of *silymarin*, an isomeric mixture of seven flavonolignans present in *milk thistle*, in a randomized controlled double-blind trial in 60 patients with type 2 DN and macroalbuminuria (urinary albumin excretion >300 mg/24 h). Administration of three daily tablets (140 mg of silymarin each) for 3 months reduced urinary albumin-creatinine ratio (UACR), urinary levels of TNF-α and urinary and serum levels of MDA. *Silymarin* also demonstrated to improve the glycemic profile (reducing fasting blood glucose and HbA1c) when administered to diabetic patients in addition to the standard treatment (hypoglycemic agent) [115,116].

Borges et al. [117] also evaluated the impact of *green tea extract* (*epigallocatechin gallate*) on the UACR in T1D and T2D patients. Administration of four daily capsules of 200 mg of *epigallocatechin gallate* to 24 patients for 3 months resulted in a significant decrease in UACR (41% of baseline). These results are in accordance with the results obtained by Teixeira [118] and Yang [119], who also observed a decrease in the UACR levels through administration of flavonoids in T2D patients. A later study performed by Sattarinezhad [120] also showed a decrease in the UACR when administering *resveratrol*. A meta-analysis performed by Liu et al. [121] showed the potential renal protective effects of *breviscapine* (flavonoid of *Erigeron breviscapus*) injections, reducing urine protein, serum creatinine and blood urea nitrogen in DN patients. Despite the progress in the flavonoids nephroprotection research, further studies in larger cohorts, longer follow-up and more robust endpoints (e.g., glomerular filtration rate and disease progression) are needed to assess the use of flavonoids as an add-on therapy to current standard of care in DN.

### 6.2. Diabetic Retinopathy

The therapeutic role of flavonoids in DR has been evaluated in several clinical trials using mainly *pycnogenol* and *diosmin*. Steigerwalt et al. [122] evaluated in 46 T2D patients with well controlled blood glucose (HbA1c <7%) and early stages of DR the efficacy of *pycnogenol* to improve microcirculation, retinal edema, and visual acuity. Subjects were daily treated with 3 × 50 mg *pycnogenol* or placebo tablets for 2 months. Visual improvement was perceived in 18 of 24 patients in the *pycnogenol* group and visual acuity significantly improved in the treated group. It was concluded that *pycnogenol* taken at early stages of retinopathy may enhance retinal blood circulation accompanied by regression of edema, which favorably improves patient vision. Forte et al. [123] evaluated, in 70 T2D patients with diabetic cystoid macular edema (CME) without macular thickening, the long-term effects of an oral pill containing 300 mg of *Diosmin*, 15 mg of *Centella asiatica* and 160 mg of *Melilotus*. They concluded that oral administration of this combination provided preservation of retinal sensitivity during the 36 months of follow up, when compared with untreated patients. Mahoney et al. [124] evaluated the influence of dietary flavonoids in data of 381 diabetic patients from the National Health and Nutrition Examination Survey (NHANES from 2003 to 2006). C-reactive protein, HbA1C, fasting glucose and insulin were measured in blood samples and a retinal imaging exam was done to assess DR. A high-flavonoid fruit and vegetable consumption index was created from a food frequency questionnaire and it was concluded that diabetic adults had lower degrees of inflammation, better glycemic control, and reduced odds of diabetic retinopathy when consuming more flavonoids in the diet. Domanico et al. [125] evaluated the changes in circulating levels of ROS and central macular thickening in 68 patients with non-proliferative diabetic nephropathy. One daily tablet containing 50 mg of *pycnogenol*, 30 mg of vitamin E and 20 mg of coenzyme Q_10_ was administered for 6 months and the blood levels of ROS were determined using the free oxygen radical test. A significant decrease in the free radical levels of the patients receiving the anti-oxidant therapy was observed, whereas a significant increase occurred in the control group. The central macular thickening was significantly reduced in the treated group, whereas no significant changes were found in the control group. A review made by Zhang et al. [126], analyzed studies published up to June 2018 that evaluated the effectiveness and safety of single herbal medicine in DR. They included randomized controlled trials and quasi-randomized trials that investigated the effects of any single herb (or extracts from a single herb) as a treatment for people with DR. The authors could not draw conclusions about the effect of any single herb or herbal extract on DR from the current available evidence due to the lack of placebo control group. Therefore, further adequately designed trials are needed to establish the evidence.

### 6.3. Diabetic Neuropathy

In diabetic neuropathy, the only flavonoids that have been studied are *quercetin* and *puerarin*. Valensi et al. [127] evaluated in a proof-of-concept study the effect of a topical compound containing *quercetin* in 34 patients. The primary objective was to decrease the oxidative stress that contributes to peripheral diabetic neuropathy and thus to alleviate the symptoms. The compound appeared to have certain effects, offering relief of diabetic neuropathy symptoms and improving quality of life. Subsequently, they performed a bigger trial [127], where this effect was further studied, but no results were published. Wu et al. [128] made a systematic review and meta-analysis of randomized controlled trials involving efficacy and safety of *puerarin* injection in treatment of diabetic peripheral neuropathy. The authors included 22 low-quality studies involving 1664 participants. *Puerarin* was effective and safe for the treatment of diabetic peripheral neuropathy. However, since the articles included in the study were not of high-quality, more studies should be conducted to strengthen their findings. Zheng et al. [129] reviewed the therapeutic efficacy of combined therapy of diabetic peripheral neuropathy with *breviscapine* and mecobalamine, an endogenous coenzyme B12. They analyzed 17 studies with a total of 1398 patients including 718 treated with *breviscapine* and mecobalamin and 680 treated with mecobalamin alone. In these studies, patients received diet control, physical exercise, and glucose-lowering therapy before interventions. This meta-analysis suggests that the combined therapy is safe and effective for these patients, although more high-quality, controlled, randomized clinical trials are needed for further confirmation.

### 6.4. Cardiovascular Complications

Early research [130,131] provided some evidence that cocoa products, which have a high content of flavonoids, may contribute to cardiovascular protection. Accordingly, a recent review by Ried et al. [132] afforded evidence for the blood pressure-lowering effect of *flavanol-rich chocolate* and *cocoa* products. Dicks et al. investigated whether regular ingestion of 2.5 g/day of flavanol-rich *cocoa powder*, in addition to their standard medication, might affect blood pressure as well as glucose and lipid metabolism in 42 stable treated subjects with T2D. They did not see any improvement in cardiometabolic parameters and attributed this finding to the fact that antihypertensive medication and *cocoa flavonols* could modulate partly the same targets. Therefore, they outlined the need of new studies focusing on the preventive effect of cocoa against diabetes and other cardiometabolic diseases in individuals with preexisting abnormalities that do not require any pharmacological treatment. Curtis et al. [133,134,135] evaluated the effect of 1 year therapy with flavonoids, in addition to statin-therapy, on cardiovascular risk reduction in postmenopausal women with T2D, and concluded that long-term *flavan-3-ol* and *isoflavone* intakes may improve markers of arterial stiffness and aspects of hemodynamic function in T2D patients taking established medication. However, additional studies are required to confirm this theory. Homayouni et al. [136] carried out a clinical trial on 64 T2D patients to study the blood pressure lowering and anti-inflammatory effects of *Hesperidin* (500 mg/day) administered for 6 weeks. Data on blood pressure and anti-inflammatory markers was recorded. The *hesperidin* group, but not the control group, had a significant reduction in systolic blood pressure and mean arterial blood pressure after the intervention period and the level of IL-6 and TNF-α were significantly reduced. The Thiazolidinediones Or Sulfonylureas Cardiovascular Accidents Intervention Trial (TOSCA.IT) study analyzed the dietary habits of 2573 participants with T2D [137]. Anthropometric measures, plasma lipids, blood pressure, C-reactive protein and HbA1c levels were measured. It was concluded that a diet characterized by a higher intake of total polyphenols was associated with a better cardiovascular risk factors profile and a lower grade of subclinical inflammation.

## 7. Ongoing Clinical Trials

A search in clinicaltrials.gov and in the *AdisInsight* database was done to analyze ongoing clinical trials related with Flavonoids and Diabetes. Only five trials were found (Table 3). Among them, the only diabetic complication approached was DN.

One of the studies [138] will evaluate the effects of *green tea extract* on RAGEs in T2D patients with DN. Thirty patients will receive 400 mg of *tea extract* or placebo twice a day during 12 weeks and soluble RAGEs and renal damage will be evaluated through the measure of the following parameters: soluble RAGE concentration, glomerular filtration rate, albumin/creatinine ratio, fasting plasma glucose, Hb1Ac concentration, systolic and diastolic arterial pressure, alanine aminotransferase, aspartate aminotransferase, total cholesterol, triglycerides, HDL-cholesterol, LDL-cholesterol, weight and visceral fat. Another study [139] evaluates the efficacy of *fisetin* on stem cells in 30 patients with diabetic kidney disease. *Fisetin* will be administered for two consecutive days vs. placebo and the efficacy on adipose tissue-derived mesenchymal stem/stromal cell function, kidney function (eGFR, UACR), markers of inflammation (C-reactive protein), and physical function in individuals with advanced chronic kidney disease will be tested. Other study is also evaluating the role of flavonoids on diabetic foot ulcers [140]. Furthermore, a 4 week interventional study is planned to evaluate the metabolic benefits of drinking three daily cups of *blueberry tea* in 36 T2D patients [141]. Also, a study to evaluate the effects of Mediterranean diet before coronary artery by-pass grafting surgery [142] is going to be performed on 48 patients with T2D.

There are also two observational studies planned [143,144], with a follow-up period higher than 10 years, that will study the appearance of different clinical conditions, for example DM and metabolic syndrome, and try to correlate them with their risk factors. In brief, although there are some ongoing clinical trials, there is still a lack of trials targeted to investigate the possible beneficial effect of different therapeutic strategies based on flavonoids in diabetic complications. Moreover, there are some clinically unexplored diabetic complications where flavonoids could be beneficial.

## 8. Toxicity and Adverse Effects of Flavonoids

Although most *flavonoids*, *flavonoid-rich foods*, *flavonoid-rich dietary patterns* and *phenolic compounds* are considered safe, there have been studies of toxic flavonoid-drugs interactions, liver failure, contact dermatitis, hemolytic anemia and estrogenic-related concerns, among others [145]. In this regard, Alkhalidy et al. mentioned some studies where flavonoids, in the presence of copper, may become pro-oxidant as well as the potential mutagenicity of *quercetin* after long-term intake [51]. The authors also suggest that flavonoids should not be given in large quantities until their biological and potential adverse effects are clarified. The studies of Bugel et al., using a zebrafish embryo-larval toxicity bioassay, also showed adverse effects on early embryogenesis of some flavonoids and flavonoid-like chemicals [146]. Therefore, the potential harmful effects of certain flavonoids should be considered by pharmaceutical companies at the time of preclinical validation both in vitro and *in vivo*.

One of the major problems of bringing flavonoids to the market is, in general, its poor availability. This information has been gathered mainly with flavonoids contained in many foods. Due to the great variability of flavonoids and phenolic compounds, each of them should be evaluated when planning its development for clinical use. Among the different strategies designed with the aim of optimizing the solubility and bioavailability of flavonoids, novel strategies are needed [147,148]. In this regard, the application of flavonoids through nanoparticles and nanodelivery has been considered in DM [149].

## 9. Future Perspectives and Conclusions

**Figure 3:**
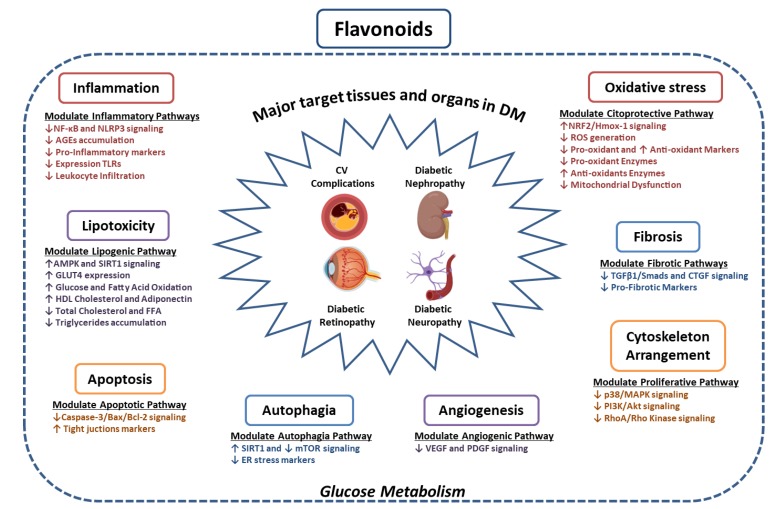
Potential beneficial effects of flavonoids in specific target organ of DM complications. AGEs, advanced glication end products; AMPK, activated protein kinase; Bax, Bcl-2 associated X; Bcl-2; B-cell lymphoma 2; CTGF, connective tissue growth factor; FFA, free fatty acid; GLUT4, glucose transporter type 4; HDL Cholesterol, High Density Lipoprotein Cholesterol; Hmox-1, Heme oxygenase (decycling) 1; MAPK, mitogen-activated protein kinases; mTOR, mammalian Target of Rapamycin; NF-Kβ, Nuclear factor kappa beta; NRF2, Nuclear Factor Erythroid 2-related Factor 2; PDGF, Platelet Derived Growth Factor; Rho, Ras homologous; RhoA, Ras homolog family member A; ROS, reactive oxygen species; SIRT1, sirtuin 1; TGFβ1, transforming growth factor beta 1; TLRs, toll like receptors; VEGF, vascular endothelial growth factor.

This comprehensive review addresses, from an objective point of view, the current state of the art about the preclinical and clinical use of flavonoids in tissue-specific manifestations of DM. As from the year 2000, the number of published studies regarding the therapeutic application of flavonoids has been exponential. There is extensive evidence of the anti-diabetic effects of flavonoids, mainly based on the local modulation of the inflammatory, oxidative and lipotoxic microenvironment leading to a protective effect in the tissue and organs affected by DM (Figure 3).

DM is a current global epidemic disease and, despite the fact that current therapies have facilitated a better control of the disease, few are the therapies aiming to regulate local inflammatory and oxidative stress. Potentially, the introduction of nutraceuticals into clinical practice could have an added value in reducing or retarding the micro or macrovascular complications associated with DM and therefore the direct and indirect health costs devoted to these patients. One of the great difficulties described for the clinical application of flavonoids refers to the low scientific evidence regarding the in vivo biodistribution and pharmacokinetic parameters of these compounds. Additionally, because the complications of DM are characterized by a slow and insidious development, the preclinical assessment of its therapeutic effects becomes even more complex.

The current technological development in the pharmaceutical industry predicts a promising future of the use of flavonoids in inflammatory based diseases, increasing the purity percentage of compounds, optimizing their formulation in order to obtain greater stabilization in vivo and target tissue bioavailability, thus prolonging their therapeutic effect.

In brief, we provide updated information concerning the discrepancy between the numerous experimental studies supporting the efficacy of flavonoids on diabetic complications and the lack of appropriate and well-designed clinical trials. Due to the well-described beneficial effects on different mechanisms involved in diabetic complications, the excellent tolerability and low cost, future randomized controlled studies with compounds that have adequate bioavailability should be evaluated as add-on therapy on well-established anti-diabetic drugs.

## Figures and Tables

**Table 1:** Effects and Mechanisms of Action of Flavonoids on Chronic Complications of DM. In vivo and in vitro Experiments.

**In vivo Studies**							
Disease	**Animal**	**DM**	**Model**	**Treatment**	**Results**	**Effect**	**PMID**
Diabetic Nephropathy	Rat	T1D	STZ	*Hesperidin* 100 mg/kg/day 2 weeks	↓FGF-23; ↑α-Klotho	Anti-toxic in liver/kidney	30551370
	Rat	T1D	Alloxan	*Ramipril* 5 mg/kg + Rutin 50 mg/kg/day 6 weeks	Prevented podocyte injury, ↓TGF-β ↓ GRP78 and CHOP (ER stress markers)	Anti-oxidant and anti-fibrotic	30372836
	Rat	T1D	STZ	*Resveratrol* 5 mg/kg/day 45/90 days	↓Renal hypertrophy and structural changes. ↓AGEs accumulation; ↓oxidative stress	Renoprotective, anti-fibrotic and anti-oxidant	29229234
	Rat	T1D	STZ	*Naringin* 20–80 mg/kg/day 12 weeks	↓ROS: ↑SOD and ↓NOX4	Renoprotective and anti-oxidant	28395989
	Rat	T2D	HFD/STZ	*Pyrrosiae folium* 50–200 mg/kg/day 12 weeks	↓ renal IL-6, TNF-α and IL-1β	Renoprotective and anti-inflammatory	29945390
	Rat	T2D	HFD/STZ	*Formononetin* 10–40 mg/kg/day. 16 weeks	↑ creatinine clearance; ↑SIRT1; ↑SOD; ↑catalase	Renoprotective, anti-diabetic and anti-oxidant	30641085
	Mouse	T1D	STZ	*Diosmetin* 25–100 mg/kg/day 8 weeks	Reduced serum FBG, BUN and creatinine and albuminuria.↓ Akt and NF-κB expression. ↑ iNOS	Renoprotective, anti-diabetic, anti-inflammatory and anti-oxidant	30278036
	Mouse	T2D	db/db mouse	*Chrysin* 10 mg/kg/day 10 weeks	↓ Collagen fiber accumulation ↓ AGEs accumulation	Anti-fibrotic	29987200
Diabetic Retinopathy	Rat	T1D	STZ	*Catechin* 50–200 mg/kg/day 8 weeks	Modulation NF-κB pathway ↓ IL-1β, IL-6, and TNF-α	Anti-inflammatory	30373863
	Rat	T1D	STZ	*Biochanin A* 10–15 mg/kg/day. 6 weeks	↓ TNFα, IL-1β and VEGF	Anti-inflammatory and anti-angiogenic	30054234
	Rat	T1D	STZ	*Trans-Resveratrol* 5 mg/kg/day. 2–4 weeks	↑ Cyp26b1 and Cyp3a9 transcription levels	Anti-oxidant	30030988
	Rat	T1D	STZ	*Morus alba extract* 100 mg/kg/day 16 weeks	↓ Caspase-3, Bax and ↑ Bcl2; ↓TNF-α and IL-1β; ↑CAT, SOD and GPx. ↓VEGF	Anti-apoptotic, anti-oxidant, anti-inflammatory and anti-angiogenic	27059477
	Rat	T1D	STZ	*Naringenin* 50 mg/kg/day 5 weeks	↑GSH; ↓Caspase-3, Bax and ↑ Bcl2; ↓pro-BDNF and ↑BDNF	Neuroprotective, anti-oxidant and anti-apoptotic	29064407
	Mouse	T1D	STZ	*Galangin* 10 mg/kg/day 30 days	↑Occludin and claudin1; ↓Iba-1 ↓ TNFα, IL-1β and IL-6 ↓p65, IκB and IKK phosphorylation	Neuroprotective and anti-inflammatory	30597356
	Mouse	T2D	db/db mouse	*Chrysin* 10 mg/kg/day 10 weeks	Increasing retinoid binding proteins (RPE65, LRAT, RDH5, and rhodopsin)	Anti-oxidant	30096827
Diabetic Neuropathy	Rat	T1D	STZ	*Catechin* 25/50 mg/kg/day 4 weeks	↑SOD and CAT ↓MDA and lymphocyte infiltration	Neuroprotective and antioxidant	30372853
	Rat	T1D	STZ	*Deguelin* 4–8 mg/kg/day 2 weeks	↑Nrf2; ↓ caspase-3 in neurons ↑ (Na+-K+) ATPase activity	Neuroprotective, anti-oxidant and anti-inflammatory	30045011
	Rat	T1D	STZ	*Resveratrol* 10 mg/kg/day 120 days	↑ SOD and GSH; ↓ nitrosative stress and MPO	Anti-oxidant and anti-inflammatory effect	29906751
	Rat	T1D	STZ	*Phloretin* 25–50 mg/kg/day 4 weeks	↑ SOD and GSH ↓ IL-6 and TNF-α; ↓ MDA	Neuroprotective, anti-oxidant and anti-inflammatory	29635891
Cardiovascular complications	Rat	T2D	HGI	*Rutin* 25–50 mg/kg/day 12 weeks	↓ inflammasome pathway in aortic tissue; ↓ROS generation	Anti-inflammatory and anti-oxidant	27936392
	Rat	T2D	HFD/STZ	*Resveratrol* 10 mg/kg/day 8 weeks	↓ TLR4/MyD88/NF-κB signaling pathway.	Cardioprotective and anti-inflammatory	30658350
	Rat	T1D	STZ	*Apigenin* 100 mg/kg/day 7 months	↓ cardiomyocyte enlargement; ↑SOD and GPx ↓ NF-κB/p65 signaling pathway activation ↓ Col-I, Col-III, CTGF, TGFβ	Cardioprotective, anti-oxidant, anti-inflammatory and anti-fibrotic	28176247
	Rat	T1D	STZ + IRIA	*Resveratrol* 5 mg/kg/day + *Glibenclamide* 5 mg/kg/day 6 weeks	↑ Kir6.2 expression (subunit of K_ATP_ channel)	Anti-arrhythmic	28176247
	Rat	T1D	STZ	*Heracleum Persicum* 100 mg/kg/day; 8 weeks	↓MDA; ↑GSH, CAT and SOD	Anti-oxidant	29726706
	Rat	T1D	STZ	*Isoquecertin* 40 mg/kg/day 45 days	↓ TG, PPL and FFA	Anti-hyperlipidemic	30817903
	Rat	T1D	STZ	*Galangin* 40 mg/kg 45 days	↓ TG, PPL, total cholesterol and FFA	Anti-hyperlipidemic	29952676
	Mouse	T2D	db/db mouse	*Scutellarin* 25–100 mg/kg 8 weeks	↑ high-density lipoprotein cholesterol ↓ TG and cholesterol	Anti-hyperlipidemic	30881587
	Mouse	T2D	HFI	*Liquiritigenin* 4–16 mg/kg/day	↓NF-κB signaling pathway ↓α-SMA, Col-I, Col-II, TGF-β1	Anti- inflammatory and anti-fibrotic	28039849
	Mouse	T2D	HFI	*Liquiritin* 10/20 mg/kg/day	↓α-SMA, Col I and Col II	Anti-fibrotic	27810791
**In vitro Studies**							
Disease	**Cell type**		**Stimulus**	**Treatment**	**Results**	**Effect**	**PMID**
Diabetic Nephropathy	Human embryonic kidney cells		HG	*Combretum micranthum* 10–25 µg/mL	Hydrogen peroxide and nitric oxide scavenging activity	Anti-oxidant	30976670
	Rat mesangial cells		HG	*Marein* 100–400 µM	Regulating AMPK, TGF-β1/Smads pathway ↓ NF-κB signaling pathway	Anti-inflammatory and anti-fibrotic	30630477
	Rat/human renal tubular epithelial cells		HG	*Kaempferol* 5–50 μM	↓ RhoA/Rho Kinase signaling	Anti-inflammatory, anti-oxidant and anti-fibrotic	30551415
	Mesangial cells		HG	*Corn silkextract* 200 µg/mL	↓ α-glucosidase and α-amylase, IL-6, AGEs, Col IV and fibronectin	Anti-inflammatory, anti-oxidant and anti-fibrotic	30530231
	Rat mesangial cells		AGEs	*Kaempferitrin* 10–35 µM	↑SOD activity ↓MDA, Col IV and TGF-β1	Anti-oxidant and anti-fibrotic	30373106
	Rat mesangial cells		HG	*Liquiritigenin* 20–40 µM	↓ NOX4 and ↑SOD Decrease collagen IV fibronectin, Il-6 and IL-1β	Anti-oxidant, anti-inflammatory and anti-fibrotic	30119269
	Human proximal tubular epithelial cells		HG	*Astilbin* 10–20 µM	Modulating PI3K/Akt pathway	Anti-proliferative	30119185
	Mouse podocyte cell line		HG	Genistein 20 µM	Inactivating mTOR signaling	Autophagia	29999001
	Human mesangial cells		HG	*Chrysin* 1–20 µM	↓ Collagens, α-SMA, fibroblast-specific protein-1, MMPs	Anti-fibrotic	29987200
	Rat mesangial cells		HG	*Naringin* 5–80 μmol/L	Modulating NLRP3 signaling pathway ↓IL-1β, IL-18 and caspase-1	Anti-inflammatory and anti-apoptotic	29929501
	Rat mesangial cells		AGEs	*Kaempferitrin* 10–35 μM	↓MDA levels; ↑SOD and ROS generation. ↓ Collagen IV and TGF-β1	Anti-oxidant and anti-fibrotic	30373106
	Human mesangial cells		AGEs	*Chrysin* 1–20 µM	↓ Collagens, α-SMA, fibroblast-specific protein-1, MMPs	Anti-fibrotic	29987200
Diabetic Retinopathy	Human retinal pigment epithelial cell line		Glucose oxidase	*Myricetin* 40 μg/mL	Activation of Nrf2 ↑ SOD ↓ NOS2	Anti-oxidant	30820141
	Human retinal pigment epithelial/endothelial cells		HG	*Baicalin* 2.5–100 μM	Inhibition of NF-κB and p38 MAPK pathways	Anti-apoptotic and anti-inflammatory	30625293
	Human retinal pigment epithelial cell line		H_2_O_2_	*Kaempferol* 20–100 nM	Modulation Bax/Bcl-2/caspase-3 pathway; ↑ SOD and ↓VEGF	Anti-apoptotic, anti-oxidant, and anti-angiogenic	30584457
	Human retinal pigment epithelium cells		HG	*Chrysin* 1–20 µM	↓VEGF and PDGF, AGEs and ER stress	Anti-angiogenic	30096827
	Human retinal capillary endothelial cells		HG	*Blueberry Anthocyanin* 10 μg/mL	↑CAT, SOD, ↓ Nox4 and eNOS levels ↓ICAM-1 and NF-κB; ↓VEGF	Anti-oxidant, anti-inflammatory and anti-angiogenic	29682153
	Rat retinal ganglial cells		HG	*Eriodictyol* 5–20 μM	↓ROS and ↑ SOD, GPx, catalase ↓ TNFα, IL-8	Anti-oxidant and anti-inflammatory	30317656
	Mice microglia retinal cells		HG	*Galangin* 20/50 µM	↓IL-1β, TNFα;↓ NF-κB activation	Anti-inflammatory	30597356
Diabetic Neuropathy	Rat dorsal root ganglion neurons		HG	*Quercetin* 10 mmol/L + other flavonoids	↑ Nrf-2/HO-1 pathway; scavenging ROS ↓NF-κB activation	Anti-oxidant and anti-inflammatory	28861887
	Schwann cells		HG	*Puerarin* 10 µmol/L	↓Caspase-3; ↓ROS production and mitochondria depolarization	Anti-apoptotic and anti-oxidant	28181078
	Rat dorsal root ganglion neurons		HG	*Quercetin* 2.5–10 mmol/L	↑ Nrf-2/HO-1; ↓ NF-κB inhibition	Anti-oxidant and anti-inflammatory	23770986
Cardiovascular complications	Human aortic endothelial cells		Palmitic acid	*Blueberry Anthocyanins* 12–16 nmol	↓Nox-mediated ROS production	Anti-oxidant	29024402
	Human umbilical vein endothelial cells		HG	*Rutin* 30–100 µM	↓Nox2 and Nox4	Anti-oxidant	27936392
	Human aortic endothelial cells		Palmitic acid	*Resveratrol* 50–100 µM	↓ ROS production via AMPK-mTOR pathway	Autophagia and anti-oxidant	30421395

**Table 2:** Summary of the Completed Clinical Trials with Flavonoids for the Treatment of DM.

	Objective to Study	Treatment	Duration	Results	PMID
**Diabetic Nephropathy**	Renoprotective effect of milk thistle extract on T2D patients with macroalbuminuria.	*n* = 30; 3 × 140 mg *silymarin* *n* = 30; Placebo	3 months	Significant decrease in UACR levels, urinary TNF-α and urinary and serum MDA in the silymarin group.	22770926 NCT01003236
	Effect of silymarin on glycemic control and body mass index in T2D patients with insulin resistance and poor glycemic control with oral hypoglycemic agents	*n* = 18; 200 mg *silymarin* + 10 mg Glibenclamide *n* = 21; Placebo + 10mg Glibenclamide	4 months	Silymarin treatment significantly reduced fasting and postprandial plasma glucose, HbA1c levels and body mass index.	17887949
	Effects silymarin administration on the glycemic state in T2D patients.	*n* = 25; 3 × 200 mg *silymarin* *n* = 26; Placebo	4 months	Decreased significantly FBG, HbA1c, total cholesterol, LDL, triglyceride, GOT and GPT after treatment with silymarin.	17072885
	Safety and effect of green tea (epigallocatechin gallate, EGCG) in patients with DN.	*n* = 24; ACEi/ARBs + 800 mg *EGCG* *n* = 23; ACEi/ARBs + Placebo	3 months	Treatment with green tea extract reduced UACR by 41%.	27320846 NCT01923597
	Effects of isolated soy protein consumption on urinary albumin excretion and blood lipid profile in early stages of DN.	*n* = 14; 0.5 g/kg/day of the dietary protein was provided as either *isolated soy protein*	2 × 8 weeks	Soy protein consumption reduced UACR levels by 9,55%.	15284369
	Efficacy of curcumin for blocking DN development in T2D patients (short time).	*n* = 14; 500 mg *curcumin*	1 month	Curcumin attenuated microalbuminuria and reduced plasma MDA and LPS levels content. Maintaining gut barrier integrity and function.	25875220
**Diabetic Retinopathy**	Effects of pycnogenol in early stages of DR.	*n* = 24; 150 mg *pycnogenol* *n* = 22; Placebo	2 months	Visual improvement was subjectively perceived by 18 of 24 patients in the pycnogenol group. Significant improvement visual acuity from baseline.	19916788
	Evaluate long-term follow-up of the orally administered combination of flavonoids for treatment of diabetic cystoid macular edema without macular thickening.	*n* = 35; 300 mg *Diosmin*, 15 mg *C. asiatica* 160 mg *Melilotus* *n* = 35; Placebo	3 years	Retinal sensitivity reduced in control group only from month 6 until month 36. In the treatment group, a greater retinal sensitivity was present at month 12, 24, and 36.	23844756
	Determine the relationship between dietary flavonoid-rich fruit and vegetable consumption on DM-related biomarkers and DR.	Data from 381 participants with DM from the NHANES 2003–2006.	-	Greater high-flavonoid fruit and vegetable consumption was associated with lower levels of CRP, HbA1c and glucose, with reducing the odds of having diabetic retinopathy by 30%.	25055729
	Efficacy of anti-oxidant dietary supplementation reducing the ROS levels in patients with non-proliferative DR.	*n* = 34; 50 mg *pycnogenol*, 30 mg Vit. E, 20 mg CoQ *n* = 34; Placebo	6 M	In the group receiving antioxidant therapy the levels of free oxygen radicals and retinal thickness were significantly reduced over three times. Conversely, in the control group a significant increase was observed.	25686055
**Diabetic Neuropathy**	Efficacy and safety of QR-333 (quercetin, ascorbyl palmitate and vitamin D3) in the treatment of diabetic neuropathy.	*n* = 23; three topical applications *QR-333* *n* = 11; Placebo	1 M	QR-333 produced significant relief of some symptoms of diabetic neuropathy and was safe and well tolerated.	16112498 NCT16112498
**Cardiovascular complications**	Effect of flavanol-rich chocolate in patients with hypertension.	*n* = 20; Cross-over 100 mg *Dark Chocolate* (88 mg flavanols) 90 mg flavanol-free White Chocolate	15 d per treatment	Dark chocolate decreased blood pressure and serum LDL cholesterol, improved FMD, and ameliorated insulin sensitivity in hypertensive patients.	16027246
	Evaluate whether regular ingestion of an unsweetened, strongly defatted and flavanol-rich cocoa powder might improve BP and glucose and lipid metabolism in stably treated T2D subjects.	*n* = 17; Five × 0.5 g *cocoa powder* capsules *n* = 18; Placebo	3 M	Daily intake of 2.5 g of flavanol-rich, unsweetened and strongly defatted cocoa powder does not affect BP, glucose and lipid metabolism in stably-treated patients with T2D and hypertension in a fasting state.	30301127
	Effect of dietary flavonoids on CVD risk in postmenopausal women with T2D on established statin and hypoglycemic therapy.	*n* = 59; 27 g flavonoid-enriched chocolate n= 59; Placebo	12 M	Improvement in insulin sensitivity was observed. Reductions in total cholesterol, HDL-cholesterol ratio and LDL cholesterol. Estimated 10 year total coronary heart disease risk was attenuated after flavonoid intervention.	22250063 NCT00677599
	Effect of combined isoflavone and flavan-3-ol intake on vascular function in postmenopausal women with T2D.			The flavonoid intervention did not significantly change the intima-media thickness of the common carotid artery, augmentation index, or BP, but pulse pressure variability improved.	
	Effect of oral Hesperidin supplementation in hemodynamic changes in T2D patients.	*n* = 32; 500 mg *Hesperidin* *n* = 32; Placebo	1.5 M	Significant difference in mean percent change of SBP, diastolic blood pressure, mean arterial BP, serum TAC, and inflammatory markers between Hesperidin and control groups.	29468764
	Association between the intake of total polyphenols and polyphenol classes with the major CV risk factors in a T2D population. TOSCA.IT study.	*n* = 2573 people with T2D	10 years	A diet characterized by a higher intake of total polyphenols was associated with a better cardiovascular risk factors profile and a lower grade of subclinical inflammation.	27890487 NCT00700856

**Table 3:** Ongoing Clinical Trials of Flavonoids, Flavonoid-Rich Foods and Flavonoid-Rich Dietary Patterns Intervention in DM.

Title	Design	Objective	Patients	Treatment	Duration
Green tea extract on Soluble RAGE in Patients with DN (NCT03622762)	Double blind, randomized and placebo controlled	Evaluate effect of administration *of green tea extract* on soluble RAGE and renal damage in patients with T2D.	30 patients	Twice daily: 400 mg green tea extract vs. 400 mg placebo	3 months
Inflammation and Stem Cells in Diabetic and CKD (NCT03325322)	Randomized, parallel	Study the efficacy of *fisetin* on stem/stromal cell function, kidney function, inflammation and physical activity in advanced CKD patients.	30 patients	Fisetin 20 mg/kg/day, orally for 2 consecutive days vs. placebo	1 year
Randomized controlled study to evaluate the efficacy and safety of WH-1 ointment for the treatment of chronic diabetic foot ulcers (NCT01898923)	Randomized, evaluator blinded, active-controlled, multicentric	Evaluate efficacy and safety of *WH-1 ointment* compared to Aquacel hydrofiber dressing.	236 patients	WH-1 ointment (1,25%) twice daily for up to 16 weeks vs. Aquacel hydrofiber dressings changed daily, on alternate days or three times a week	4 months
Metabolic Benefits of Drinking Blueberry Tea in T2D (NCT02629952)	Open, randomized, crossover assigned.	Determine whether chronic consumption of *blueberry tea* improve metabolic and vascular health in people with and without T2D.	36 patients	Three cups of blueberry tea per day vs. non treatment	1 month
Effects of Mediterranean Diet Intervention in Diabetic Heart Disease (NCT03757845)	Randomized, parallel, double-blind	Examine short-term effects of modified Mediterranean diet on lipogenic signaling pathway in T2D patients.	48 patients	Mediterranean diet vs. control diet	9 days

## Abbreviations

α-SMA, alpha-smooth muscle actin; ACE, angiotensin converting enzyme; BDNF, brain-derived neurotrophic factor; CAT, catalase; Col I, collagen I; Col II, collagen II; Col IV, collagen IV; eNOS, endothelial nitric oxide synthase; ER, endoplasmic reticulum; FFA, free fatty acids; FGF-23, fibroblast growth factor 23; GPx, glutathione peroxidase; GSH, glutathione; HFI, High fructose induce model; HG, high glucose; HO-1 Heme oxygenase 1; HPE, H. sabdariffa (roselle) polyphenol-rich extract; IBA1, ionized calcium-binding adapter molecule 1; ICAM-1, Intercellular Adhesion Molecule 1 IL-6, interleukin 6; IL-18, interleukin 18; LC3-II, LC3-phosphatidylethanolamine conjugate; LRAT, lecithin retinol acyltransferase; MAPK, mitogen-activated protein kinases; MDA, malondialdehyde; MPO, myeloperoxidase; NF-κβ, nuclear factor κβ; NOX4, NADPH oxidase 4; Nrf2, nuclear factor erythroid 2-related factor 2; PEDF, and pigment epithelium-derived factor; PI3K, phosphoinositide 3-kinase; PPL, phospholipids; RDH5, retinol dehydrogenase 5; ROS, reactive oxygen species; RPE65, retinal pigment epithelium-specific 65 kDa protein; SIRT1, sirtuin 1; SOD, superoxide dismutase; T2D, type 2 diabetes; T1D, type 1 diabetes; TG, triglycerides; TGF-β1, transforming growth factor beta 1; TNFα, tumor necrosis factor alpha; VEGF, vascular endothelial growth factor.
